# Correction: Single-cell RNA sequencing of human breast tumour-infiltrating immune cells reveals a γδ T-cell subtype associated with good clinical outcome

**DOI:** 10.26508/lsa.202201848

**Published:** 2022-12-13

**Authors:** Katerina Boufea, Victor Gonzalez-Huici, Marcus Lindberg, Nelly N Olova, Stefan Symeonides, Olga Oikonomidou, Nizar N Batada

**Affiliations:** 1 Centre for Genomic and Experimental Medicine, University of Edinburgh, Edinburgh, Scotland; 2 MRC Human Genetics Unit, University of Edinburgh, Edinburgh, Scotland; 3 Cancer Research UK Edinburgh Centre, University of Edinburgh, Western General Hospital, Edinburgh, Scotland

## Abstract

Single cell sequencing of γδ-T cells from human blood and tumours revealed novel markers of subtypes with distinct effector functions and a subtype that is associated with favourable clinical outcome.

Article: Boufea K, Gonzalez-Huici V, Lindberg M, Olova NN, Symeonides S, Oikonomidou O, Batada NN (2020 Dec 2) Single-cell RNA sequencing of human breast tumour-infiltrating immune cells reveals a γδ T-cell subtype associated with good clinical outcome. Life Sci Alliance 4(1): e202000680. doi: 10.26508/lsa.202000680. PMID: 33268347.

In the first published version of this paper, Nelly N Olova was omitted from the author list. She has contributed with 10× Chromium single-cell library preparation. All authors are mentioned above in the corrected order. The authors apologize for the error, which has now been fixed in the online version of the article.

## Supplementary Material

Reviewer comments

