# Supplementary material for: Correction: Single-cell RNA sequencing of human breast tumour-infiltrating immune cells reveals a γδ T-cell subtype associated with good clinical outcome
Source: Life Sci Alliance. 2022 Dec 13;6(2):e202201848. doi: 10.26508/lsa.202201848 (PMC9748721; doi:10.26508/lsa.202201848)
Supplement: Reviewer comments [file LSA-2022-01848_review_history.pdf]

Life Science Alliance

Correction: Single-cell RNA sequencing of human breast tumour-infiltrating immune cells reveals...

Nizar Batada, Katerina Boufea, Victor Huici, Marcus Lindberg, Nelly Olova, Stefan Symeonides, and Olga Oikonomidou
DOI: https://doi.org/10.26508/lsa.202201848

Corresponding author(s): Nizar Batada, University of Edinburgh

Review Timeline:

Submission Date:	2022-11-23
Accepted:	2022-11-28

Scientific Editor: Eric Sawey, PhD

Transaction Report:

(Note: With the exception of the correction of typographical or spelling errors that could be a source of ambiguity, letters and reports are not edited. The original formatting of letters and referee reports may not be reflected in this compilation.)

November 28, 2022

RE: Life Science Alliance Manuscript #LSA-2022-01848

Dr. Nizar N Batada
University of Edinburgh
Institute of Genetics & Molecular Medicine
Crewe Road S
Edinburgh EH4 2XU
United Kingdom

Dear Dr. Batada,

Thank you for submitting your Correction entitled "Correction: Single-cell RNA sequencing of human breast tumour-infiltrating immune cells reveals a γδ T-cell subtype associated with good clinical outcome".

IMPORTANT: If you will be unreachable at any time, please provide us with the email address of an alternate author. Failure to respond to routine queries may lead to unavoidable delays in publication.

You can contact the journal office with any questions, contact@life-science-alliance.org

Sincerely,

Eric Sawey, PhD
Executive Editor
Life Science Alliance
http://www.lsajournal.org

November 28, 2022

RE: Life Science Alliance Manuscript #LSA-2022-01848

Dr. Nizar N Batada
University of Edinburgh
Institute of Genetics & Molecular Medicine
Crewe Road S
Edinburgh EH4 2XU
United Kingdom

Dear Dr. Batada,

Thank you for submitting your Correction entitled "Correction: Single-cell RNA sequencing of human breast tumour-infiltrating immune cells reveals a γδ T-cell subtype associated with good clinical outcome".

IMPORTANT: If you will be unreachable at any time, please provide us with the email address of an alternate author. Failure to respond to routine queries may lead to unavoidable delays in publication.

You can contact the journal office with any questions, contact@life-science-alliance.org

Sincerely,

Eric Sawey, PhD
Executive Editor
Life Science Alliance
http://www.lsajournal.org
